# Identification of protein targets in cerebral endothelial cells for brain arteriovenous malformation (AVMs) molecular therapies

**DOI:** 10.1186/s12014-017-9151-3

**Published:** 2017-05-16

**Authors:** Margaret Simonian, Rachel R. Ogorzalek Loo, Nalaka Rannulu, Joseph A. Loo, Mark P. Molloy, Marcus A. Stoodley

**Affiliations:** 1 Department of Clinical Medicine, Faculty of Medicine and Health Sciences, Macquarie University, North Ryde, NSW 2109 Australia; 2 David Geffen School of Medicine, Department of Biological Chemistry, University of California Los Angeles (UCLA), 611 Charles E. Young Drive East, Los Angeles, CA 90095 USA; 3 Australian Proteome Analysis Facility (APAF), Department of Chemistry and Bimolecular Sciences, Macquarie University, North Ryde, NSW 2109 Australia

**Keywords:** Endothelial cells, Biotinylation, Membrane proteins, Irradiation, Arteriovenous malformations

## Abstract

**Background:**

To develop a new molecular targeted treatment for brain (AVMs), identification of membrane proteins that are localised on the AVM endothelium is crucial. Current treatment methods are surgery and radiosurgery. However, complete occlusion post radiosurgery are achieved within 3 years, while patient remain at risk of haemorrhage. This study aims to identify potential protein targets in AVM endothelial cells that discriminate these vessels from normal vessels; these proteins targets will be investigated for the molecular therapy of brain AVMs to promote rapid thrombosis after radiosurgery.

**Methods:**

We employed in vitro biotinylation that we developed, and mass spectrometry to detect cell surface-exposed proteins in cultures of murine cerebral endothelial cells (bEnd.3). Two forms of mass spectrometry were applied (iTRAQ-MS and MS^E^) to identify and quantify membrane protein expression at various time-points following irradiation which simulates a radiosurgical treatment approach. Immunocytochemistry was used to confirm the expression of selected membrane proteins. ProteinPilot V4.0 software was used to analyse the iTRAQ-MS data and the MS^E^ data was analysed using ProteinLynx Global Server version 2.5 software.

**Results:**

The proteomics data revealed several differentially expressed membrane proteins between irradiated and non-irradiated cells at specific time points, e.g. PECAM-1, cadherin-5, PDI, EPCR and integrins. Immunocytochemistry data confirmed the expression of these proteins.

**Conclusion:**

Cell surface protein biotinylation and proteomics analysis successfully identified membrane proteins from murine brain endothelial cells in response to irradiation. This work suggests potential target protein molecules for evaluation in animal models of brain-AVM.

**Electronic supplementary material:**

The online version of this article (doi:10.1186/s12014-017-9151-3) contains supplementary material, which is available to authorized users.

## Background

Brain arteriovenous malformations (AVMs) consist of a tangle of abnormal arteries and veins linked by one or more fistulae [8]. The cause of brain AVMs is not well known, with some theories suggesting that they may be caused by a clot or a rupture of blood vessels during fetal development [23], while others suggest that they develop postnatally, undergoing a period of growth in childhood or early adulthood and that the growth may be caused by endothelial shear stress that stimulates growth factor expression [18].

Patients with AVMs present with headaches, seizures, or, most commonly hemorrhage. Current treatment options for brain AVMs are surgery, embolization and stereotactic radiosurgery. The goal of AVM treatment is to prevent haemorrhage and the choice of treatment depends on many factors, including AVM location (eloquent or non-eloquent brain) and size [8, 38, 39].

Stereotactic radiosurgery is a procedure that delivers a single, localized, high dose of radiation to the target through the intact skull using a linear accelerator (LINAC) or Gamma Knife [21, 27]. This treatment is suitable for lesions <3 cm in diameter and located in eloquent areas where surgery can cause neurological deficits [8]. However, vascular occlusion after radiosurgery can take up to 3 years to complete, and patients remain at risk of haemorrhage during this time [9, 22]. Approximately one-third of AVMs are unsuitable for current treatment methods, therefore there is a need for new treatments, especially for large and deep lesions [7, 14].

We have been investigating endothelial expressed molecules as targets for AVM molecular therapy. Specifically in this study, it is proposed that radiosurgery can modify the expression of endothelial cell surface discriminating proteins and hence provide a molecular targeting site for delivering secondary agents (e.g. pro-thrombotic molecules). To achieve this goal, we have developed in vitro biotinylation methodology to label endothelial cell surface proteins [35, 36], and we employed that methodology here to identify protein targets using two advanced quantitative mass spectrometry techniques, iTRAQ-MS and MS^E^.

MS^E^ is a label free quantitative MS technique, while iTRAQ-MS is a labelled-based proteomics method. MS^E^ enables the identification as well as quantification of proteins, improves sequence and proteome coverage, and has lower false positive rates. These advantages are most dramatic for the low abundant proteins such as endothelial membrane proteins. Therefore we used these two proteomics methods to validate our proteomics data and to increase the number of membrane proteins identification.

Mouse brain endothelial cell cultures (bEnd.3) were studied at 6, 24, 48 and 72 h post irradiation, while non-irradiated cells were served as controls. Eight-plex iTRAQ-MS and MS^E^ analyses were carried out to compare the differences in protein expression between irradiated and control samples at each time point. Immunocytochemistry was subsequently used to confirm the expression of these proteins.

## Methods

### Mouse endothelial cell cultures (bEnd.3)

Cryopreserved bEnd.3 cells obtained from (American Type Culture Collection, VA, USA) were cultured in DMEM with 4.5 g/L d-glucose, 4 mM l-glutamine, and 0.11 g/L sodium pyruvate (Gibco, CA, USA). 10% fetal bovine serum albumin (Invitrogen, Gibco), HEPES (Sigma, Aldrich, MO, USA) and antibiotics (Invitrogen, Gibco) were added to the DMEM and incubated in a 5% CO_2_ atmosphere at 37 °C. Cells were seeded in 75 cm^2^ tissue culture flasks, 15–17 mL of the growth media were added until about 80% confluent with medium renewal every 2–3 days.

### Cell density and total protein concentration

bEnd.3 cells were counted using Bio-Rad Automated Cell Counter TC10 (Bio-Rad, Castle Hill, NSW, Australia). Cells viability was assessed with Trypan Blue Solution (0.4%) (Sigma Aldrich, MO, USA). Equal amounts of cells were seeded in each flask. The total protein concentration of cell cultures was determined using Micro BCA kit (Pierce, IL, USA). A standard curve was generated by using bovine serum albumin. The density of bEnd.3 cells was approximately 1 × 10^5^ cells/mL and total protein concentration was 1.6 mg/mL.

### Irradiation of bEnd.3 cells

bEnd.3 cells were irradiated once they reached 80% confluence in their culture flasks and samples collected at 6, 24, 48 and 72 h post irradiation. Non irradiated cells were also collected at the corresponding time points. The irradiation dose used in this study was 25 Gy, which is the same dose currently are being used for stereotactic radiosurgery treatment for brain AVMs. The cells used for the iTRAQ study were irradiated at Macquarie University Hospital using 6 MV photons on an Elekta Synergy linear accelerator, and the cells used for the MS^E^ study were irradiated at UCLA Radiation Oncology department using RS320 research system (Varian Medical Systems) that uses a metal ceramic 300 kV X-ray tube with an integral high voltage receptacle and cooling system. The system is enclosed in a ray proof housing that contains fittings for water hose connections. The X-ray tube output limits are; Voltage 30–310 kV, Current 1.0–30 mA, Power 3000 W. Cells were returned to the incubator immediately after irradiation. None-irradiated cells were used as controls at each time point.

### In vitro biotinylation of bEnd.3 cells

Surface biotinylation was performed on irradiated and control bEnd.3 cultures using our developed protocol [35]. Briefly, each 75 cm^2^ flask containing approximately 1 × 10^6^ cells was washed four times with PBS pH 7.4. Twenty millilitres of PBS containing 67 µM Sulfo-NHS-LC-Biotin (Pierce, IL, USA) were added to the flasks and incubated for 5 min at room temperate. The biotinylation reaction was terminated by adding Tris–Hcl pH 7.5 to reach a final concentration of 670 µM. After 5 min incubation the cells were washed four times with PBS and harvested with 2–3 mL of lysis buffer containing 2% w/v NP40, 0.2% w/v SDS and protease inhibitor (Complete, EDTA-free, Roche, Switzerland) and kept on ice for 30 min.

### Capture of biotinylated proteins

Streptavidin Sepharose high performance (GE health care, Australia) was used to capture biotinylated proteins, according to the protocol of Simonian et al. [35]. Five hundred microlitres of streptavidin Sepharose were washed first three times with buffer A containing (1% w/v NP40, 0.5% w/v SDS in PBS). Samples were then incubated with washed streptavidin Sepharose for 2 h in room temperature. Streptavidin Sepharose was pelleted by centrifugation at 1600*g* for 5 min. Unbound proteins were removed by washing three times with buffer A, once with buffer B (0.1% w/v NP40, 0.5 M NaCl in PBS) and once with digestion buffer (0.25 mM TEAB) for iTRAQ-MS analysis. For MS^E^ analysis unbound proteins were removed by washing three times with 1% v/v TX-100, once with 0.1% w/v SDS and five times with digestion buffer (50 mM ammonium bicarbonate). The use of high salt concentration and NP40 detergent in the washing buffers, will minimise the non-specific interactions of biotin and streptavidin.

### Tryptic digestion of biotinylated proteins and iTRAQ labelling

Streptavidin Sepharose was re-suspended in 200 µL of digestion buffer. Twenty microlitres of trypsin were added and incubated overnight at 37 °C. The samples were centrifuged at 14,100*g* for 2 min at room temperature. Supernatant was removed and dried in the SpeediVac until complete dryness. Samples were resuspended in 0.5 M TEAB and labelled with iTRAQ 8-plex reagents kit (Applied Biosystems, Foster City, CA) as follows [Sample (6) = 113, control (6) = 114, sample (24) = 115, control (24) = 116, sample (48) = 117, control (48) = 118, sample (72) = 119, control (72) = 121].

### Strong cation exchange chromatography and Nano-LC ESI MS/MS

iTRAQ labelled samples were pooled in a 1:1 ratio and fractionated by strong cation exchange chromatography (SCX) using Macro-Prep High S Ion Exchange Support (Bio-rad, Cat# 158-0030) per the manufacturer’s instructions and the cleaned sample was collected and dried. The cleaned SCX fraction was resuspended in 90 µL of desalting solution containing 0.1% trifluoroacetic acid and 2% acetonitrile 97.9% water. Thirty-nine microliters of the resuspended solution was loaded on a reverse phase peptide Captrap (Michrom Bioresources) then desalted with the desalting solution at a rate of 10 µL per min for 13 min. The trap was switched on line with a 150 µm × 10 cm C18 3 µm 300A ProteCol column (SGE). The buffer solution A contained 99.9% water, 0.1% formic acid and buffer solution B was increased from 5 to 90% in 120 min in three linear gradient steps to elute the peptides. The column was then cleaned with 100% buffer B for 15 min and equilibrated with buffer A for 30 min. The reverse phase nano LC eluent was subject to positive ion nanoflow electrospray analysis. In IDA (information dependent acquisition) mode a TOFMS scan was acquired (*m/z* 380–1600 for 0.5 s), with the three most intense multiply charged ions (with counts >70), then subjected to MS/MS analysis. MS/MS spectra were gathered for 2 s in the mass range of *m/z* 100–1600 with a modified (Enhanced All Q2) transition setting to favour low mass ions so that the reporting iTRAQ tag ion (113, 114, 115, 116, 117, 118, 119 and 121) intensities were enhanced for quantitation (Australian Proteome Analysis Facility, APAF protocol).

### Chromatographic separation and MS^E^ analysis

To support the iTRAQ-MS analysis we carried out an independent MS experiment using a label-free method known as MS^E^. Cultured cells were treated by irradiation and samples collected after 6, 24 and 48 h. Control samples were also collected at these timepoints.

Chromatographic separation of the tryptic peptides was achieved using an ultra-performance liquid chromatography (UPLC) system (Waters nanoAcquity UltraPerformance UPLC) coupled to a Waters Xevo quadrupole time-of-flight mass spectrometer. Peptides were separated with a UPLC BEH C18 Column (1.7 µm, 75 µm × 150 mm, 10 K psi). The mobile phase, used at a flow rate of 0.3 µL/min, with a gradient of a mixture of (A) 0.1% formic acid in water and (B) 0.1% formic acid in acetonitrile was programmed as follows: initial 97% A for 1 min, decreased to 60% A in 60 min, then decreased to 5% for 2 min, held at this for 15 min, again increased to 97% A in 3 min. The column temperature was set at 28 °C.

Mass spectrometry analysis was performed utilizing a Waters Xevo quadrupole time of flight (Q-TOF) micro™ mass spectrometer coupled directly to Waters nanoACQUITY UPLC system (Waters Corp). All analysis was performed using positive mode electrospray ionization (ESI). The LC-mass spectrometer was operated in the MS^E^ data independent acquisition mode. LC MS data was collected in an alternating low energy MS and elevated energy MS/MS (MS^E^) mode of acquisition. In low energy MS mode the data were collected at a constant collision energy of 6 eV. In elevated energy MS/MS mode the collision energy was ramped from 15 to 40 eV on laboratory frame energy to collect product ions of all precursors identified from the MS scan.

### Immunocytochemistry

The following protocol was optimised on bEnd.3 cells then used. Cells were grown on cover slips in six well cell culture plates (CELLSTAR, Indiana, USA), and fixed with 4% paraformaldehyde for 6 min cell were washed with PBS three times and permeabilized with 0.2% (v/v) Tween-20 in PBS for another 6 min. After washing with PBS X3 and 1% (w/v) bovine serum albumin three times, cells were stained for anti-cadheren 5 (CD144), anti-CD109 (BD Pharmingen) and anti-protein disulfide isomerise (PDI) (Sigma) antibodies. The corresponding secondary antibodies were labelled with Alexa Fluor 488 (Invitrogen) and nuclei were stained with DAPI (Invitrogen). Slides were then mounted with Fluoromount mounting medium (DAKO) and examined with fluorescent microscope (Leica, Microsystems, Germany). Cells were stained at 6, 24, 48 and 72 h post irradiation. Control cells were also stained at each time point.

### Data analysis

#### iTRAQ-MS

The nano-LC ESI MS/MS data were submitted to ProteinPilot V4.0 software (AB Sciex) for data processing using SwissProt database and *Mus musculus* species. Bias correction was selected. The detected protein threshold (unused ProtScore) was set as >1.3, better than 95% confidence with *p* values <0.05. The search parameter settings were: enzyme, trypsin; maximum missed cleavages, 1; fixed modification: carbamidomethyl (Cys), iTRAQ (N terminus), iTRAQ (Lys), variable modifications, phosphorylation (Ser, Thr, Tyr) and oxidation (Met); peptide tolerance, 65 ppm; MS/MS tolerance, 0.15 Da. The number of proteins in the database was 16,307. The hypothesis being tested for ProteinPilot software was “The actual protein ratio is 1 and the observed protein ratio is different than 1 by chance.” This null hypothesis is not true when the difference in expression is real. That is, the difference between the observed ratio and 1 is due to something real, not random variation. The smaller the *p* value, the more likely that expression difference is real.

#### MS^E^

The LC MS and LC MS/MS data were processed using ProteinLynx Global Server (PLGS) version 2.5 (Waters Corporation). The quantification of protein levels was achieved by the addition of an internal protein standard (BSA trypsin digest) to which the data set was normalized. The protein identification was based on MS/MS peak lists which were generated by MS^E^ data independent collision induced fragmentation using a *Mus musculus* database. Protein identifications were accepted with greater than three fragment ions per peptide, seven fragment ions per protein and one unique peptide per protein identified. Carbamidomethyl cysteine was set as a fixed modification while oxidized methionine was set as a variable modification. Trypsin was set as a proteolytic enzyme, and up to two missed cleavages were allowed. Peptide tolerance set at 10 ppm with fragment ion tolerance of 0.5 amu.

When a peptide was not detected in the MSE experiment a nominal amount of 0.01 was reported to avoid zeros in subsequent calculations.

## Results

### Proteomics

#### iTRAQ-MS analysis

The proteomics quantitative analysis of 8-plex iTRAQ-MS was carried out with three independent biological replicates. From the first iTRAQ-MS experiment, 102 proteins were identified from a total of 3828 spectra. The second iTRAQ-MS identified 132 proteins from a total of 3938 spectra, and the third iTRAQ-MS, identified 83 proteins from a total of 5282 spectra. A total of 50 membrane proteins were identified from all three iTRAQ-MS experiments. Additional file 1: Table S1 contains the names of these proteins, and Additional file 2: Table S2 is the raw data for one of iTRAQ-MS run.

Our proteomics analysis focused on the proteins that were present and showed differences in expression level between irradiated and control samples in at least two out of the three independent iTRAQ-MS runs for statistical significance analysis. Eleven proteins were significantly differentially expressed in at least two out of the three iTRAQ-MS runs at different time points. At 6 h after irradiation, filamin B, protein disulfide isomerase, and vimentin were up-regulated in irradiated cells. At 24 h, myosin was up-regulated in irradiated cells, while at 48 h, lamin, plectin, vimentin, actin cytoplasmic 2 and histone H2A were up-regulated in irradiated cells and at 48 h, plectin, vimentin, myosin and histone H4 were up-regulated in irradiated cells at 72 h.

The membrane proteins that were up-regulated in irradiated samples, compared to the controls at different time points, are of interest in this study since those on the surface of endothelial cells potentially can be targeted by ligands to induce thrombosis in AVM vessels post radiosurgery. We found six up-regulated membrane proteins at different time points, however, some membrane protein up-regulation, although significant at their MS run (*p* < 0.05), was only observed in one out of the three iTRAQ-MS runs.

#### MS^E^ analysis

The MS^E^ experiment was repeated so that mass spectrometry data were independently obtained twice. A total of 36 mass spectrometry runs were analysed from 6 irradiated and 6 control samples, each run in triplicate. The total number of proteins identified in bEnd.3, by two independent MS^E^ analyses in irradiated (R) and control (C) samples at each time point were as follows; at 6 h, 163 proteins in (R) and 229 in (C): at 24 h, 407 proteins in (R) and 371 in (C): at 48 h, 216 proteins in (R) and 276 in (C). While the number of membrane proteins were 10 in (R) and 27 in (C) at 6 h; 47 proteins in (R) and 31 in (C) at 24 h; 18 proteins in (R) and 13 in (C) at 48 h post-irradiation, making a total of 146 membrane proteins.

Our proteomics analyses focused on the membrane proteins that were present in both independent MS^E^ data at three time points. Table 3 indicates the membrane proteins shared between irradiated (R) and control (C) samples at each time point with their average concentrations on column, average protein masses and number of times identified in the runs. The Additional file 3: Table S3 includes all the proteins identified in both MS^E^s while Additional file 4: Table S4  shows the raw data for one of the fractions of control sample at 6 h time point.

Further analysis was focused on the membrane proteins that were present in at least three out of the six MS^E^ runs in R and C groups at each time point. This criterion was observed only at 24 and 48 h time points, and is presented in (Tables 4, 5).

**Fig. 1:**
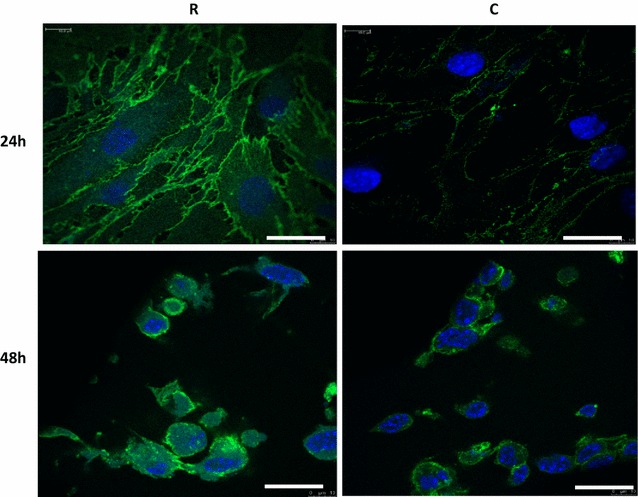
Fluorescent staining of cadherin 5 at 24 and 48 h post irradiation. Expression levels of this protein were higher in irradiated cell compared to the controls

Cadherin 5 (CD144) was expressed on the cell membrane of irradiated and non-irradiated cell cultures. The expression intensity in the irradiated cells at 24 and 48 h was greater than controls (Fig. 1). This observation was in agreement with iTRAQ-MS data at 24 h but not at 48 h (Table 2), and was in agreement with MS^E^ data at 24 and 48 h (Table 3). A change in cell shape was also noted at 48 and 72 h. The Additional file 5: Table S5, combine Tables 1, 2, 3, 4, 5 & the staining data in one table.

#### Platelet endothelial cell adhesion molecule-1

**Fig. 2:**
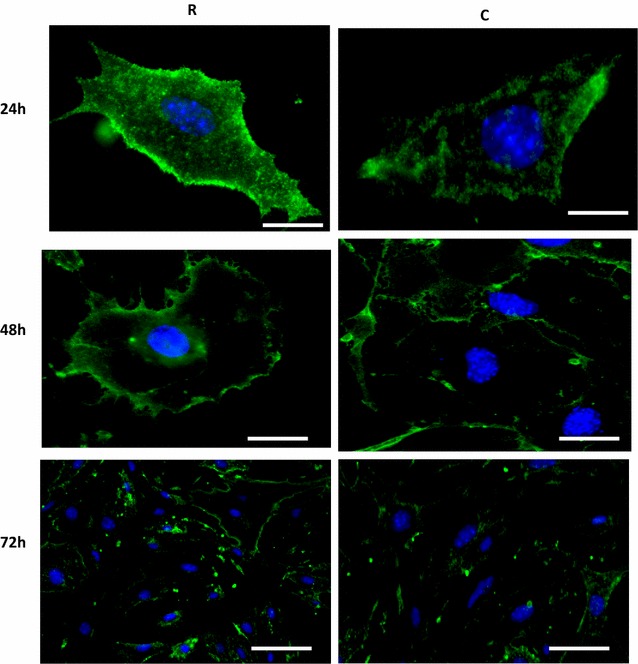
Fluorescent staining of PECAM-1 at 24, 48 and 72 h post irradiation. Expression levels of this protein were higher in irradiated cells compared to the controls at these time points

PECAM-1 expression intensity on the cell membrane was higher in irradiated cells at 24, 48 and 72 h post-irradiation compared to the control cells, however the intensity levels decreased gradually at 48 and 72 h in irradiated cells (Fig. 2). The proteomics data at 24 and 72 h are in agreement with the staining data that show elevated PECAM-1 after irradiation (Table 3). Morphological changes also noted after 24 h of irradiation, this may due to the long term effect of irradiation on cells viability.

#### CD109

**Fig. 3:**
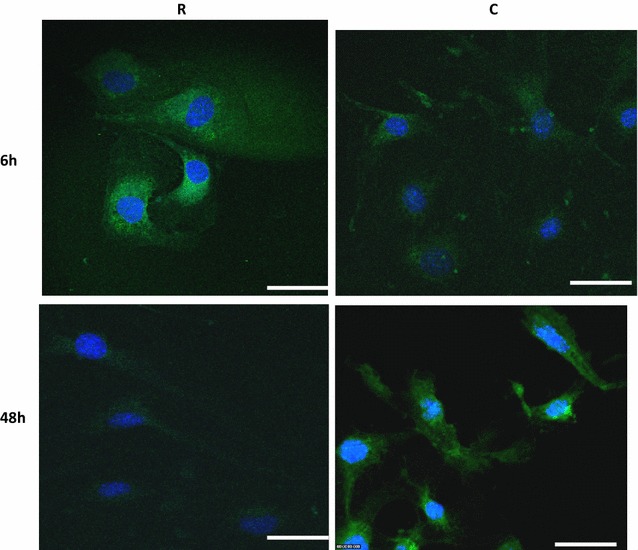
Fluorescent staining of CD109 at 6 and 48 h post irradiation. Expression levels of this protein increased at 6 h in irradiated cells compared to the controls and decreased at 48 h in irradiated cells

The CD109 was mainly expressed in the cytoplasm of irradiated and control cells. The expression intensity in the irradiated cells was higher at 6 h compared to the control, but lower at 48 h compared to the control (Fig. 3). This was consistent with iTRAQ-MS (Table 3).

#### Protein disulfide isomerise (PDI)

**Fig. 4:**
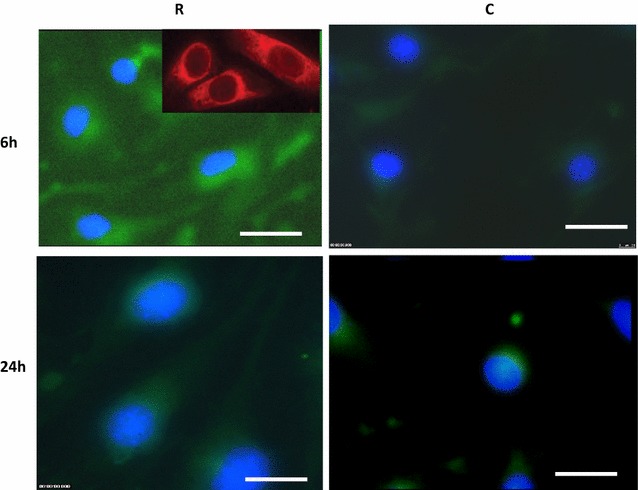
Fluorescent staining of PDI at 6 and 24 h post irradiation. Expression levels of this protein increased at 6 and 24 h in irradiated cells compared to the controls. An *image* of PDI stained with cy5 at 6 h in irradiated cells is also shown

PDI was expressed in the cytoplasm of irradiated and control cells. The expression intensity in the irradiated cells was greater in the irradiated cells than in controls at 6, 24 and 72 h time points (Fig. 4). These data are in agreement with iTRAQ-MS data at those times (Table 3). Most irradiated cells died after 72 h.

## Discussion

The use of iTRAQ-MS and MS^E^ enabled the examination of protein expression changes in irradiated murine endothelial cell cultures over a time course of up to 72 h. The aim of our study was to identify potential protein targets in AVM endothelium that discriminate these vessels from normal vessels; these proteins will be investigated for the molecular therapy of brain AVMs to promote rapid thrombosis after radiosurgery.

In general, the proteomics investigation of bEnd.3 cell cultures revealed that numerous membrane proteins were differentially expressed between irradiated and non-irradiated cells. Many of those were up-regulated in the irradiated cells, especially 24 h post-irradiation. The most significant were PECAM-1, cadherin 5, PDI, integrin alpha 5, integrin alpha 6, integrin beta 1, CD109, EPCR and multimerin 2. The up-regulation of four of these proteins was confirmed by immunocytochemistry.

### iTRAQ-MS data

Eleven proteins were significantly differentially expressed in at least two out of the three iTRAQ-MS analyses at different time points, some were membrane proteins and some not, but mostly were up regulated in the irradiated cells (Table 1). Our primary focus was on the up-regulated membrane proteins in the irradiated cells at different time points and those were PECAM-1, Cadherin-5, PDI, CD109, integrin alpha-5 and integrin alpha-6 (Table 2).

**Table 1:** Average protein expression ratios (control: irradiated), in at least two out of three iTRAQ experiments and the time point at which they showed significant up or down regulation or (fold change) with *p* < 0.05

Protein	Ave (R:C) 6 h	Ave (R:C) 24 h	Ave (R:C) 48 h	Ave (R:C) 72 h
Cadherin-5^a^	1.00	1.00	0.80	1.00
CD109^a^	1.00	1.00	0.66	1.00
Protein disulfide Isomerise (PDI)^a^	1.40	1.00	1.00	1.00
Actin cytoplasmic 2	1.00	1.00	0.81	1.00
Filamin B	1.26	1.00	1.00	1.00
Lamin	1.00	1.00	1.53	1.00
Plectin	1.14	1.00	1.28	1.18
Vimentin	1.36	1.00	2.50	1.17
Myosin	1.00	1.25	1.00	1.28
Histon H2A	1.00	1.00	3.10	1.00
Histon H4	1.00	1.00	1.00	0.42

^a^Membrane proteins

**Table 2:** Up-regulated membrane proteins of bEnd.3 cells at various time points and their average (irradiated: control) ratios, *p* < 0.05

Protein name	(R:C) 6 h	(R:C) 24 h	(R:C) 72 h
Cadherin-5	1.00	1.60	1.00
PECAM-1	1.00	1.45	1.17
Protein disulfide isomerase (PDI)	1.40^a^	1.45	1.20
CD109	1.28	1.00	1.00
Integrin alpha-5	1.21	1.41	0.87
Integrin alpha-6	1.00	1.37	1.00

^a^Average of 2/3 iTRAQ-MS

 PECAM-1 (or CD31) was up-regulated in irradiated cells at 24 and 72 h by 1.45- and 1.17-fold respectively. PECAM-1 is a vascular endothelial cell adhesion molecule that makes up the majority of endothelial cell intercellular junctions [41]. It is also expressed in platelets, macrophages and lymphocytes, and plays important roles in angiogenesis, regulation of integrin-mediated cell adhesion, and thrombosis [24].

PECAM-1 is a diagnostic membrane protein used to demonstrate the presence of endothelial cells in histological tissues [40, 42]. It is also expressed in certain tumours, which can imply a rapidly growing tumour because of its involvement in angiogenesis, for this reason PECAM-1 has been used as an angiogenic target in cancer therapies [5, 25]. Previous in vitro studies in human and animal cell cultures showed that PECAM-1 expression increased when endothelial cells were exposed to irradiation doses from 10 to 20 Gy [11].

Cadhrein-5 (CD144) is another vascular endothelial cell adhesion molecule, belonging to the family of cadherins. Cadherin 5 is required for maintaining a restrictive endothelial barrier and is essential for proper vascular development [3]. Previous studies on cadherin 5 response to irradiation showed similar expression to that of PECAM-1 [1]. Our data are in agreement with those studies, as cadherin 5 expression was up-regulated in irradiated cells by 1.6-fold at 24 h of irradiation.

Protein disulfide isomerase (PDI), showed up-regulation in irradiated cells at 6, 24 and 72 h by 1.40-, 1.45- and 1.20-fold, respectively (Table 2). PDI is an enzyme that acts on the cell surface as a reductase to cleave the disulfide bonds of proteins attached to the cell, while inside the cell it rearranges the disulfide bonds of newly forming proteins [43]. PDI is also found on the surface of platelets where it plays a role in the activation of intergrins that involves rearrangement of disulfide bonds mediated by PDI; this activation of integrins to the ligand binding state is an important part of platelet adhesion and secretion [28]. No previous evidence is available on the effect of irradiation on PDI expression in endothelial cells.

Integrins belong to a large family of transmembrane cell adhesion receptors that mediate interactions between adhesion molecules on adjacent cells and on the extracellular matrix. They exist as heterodimers, alpha and beta subunits, and play different roles in biological processes such as cell migration, wound healing and apoptosis [28, 45]. Integrin alpha 5 is a fibronectin receptor, and integrin alpha 6 is a receptor for laminin which is a major protein in the basal lamina [19, 44]. Alpha-5 beta-1 integrin mediates the adhesion pathway employed by platelets in the presence of PDI enzyme that facilitate the disulfide exchange on the cell-surface receptors [6, 12]. In our iTRAQ-MS study, the expression levels of integrin alpha 5 increased in irradiated cells at 6 and 24 h by 1.21- and 1.41-fold respectively, while integrin 6 expressions increased in irradiated cells at 24 h by 1.37-fold, making them interesting targets for further investigation for the AVM therapy post radiosurgery.

 A number of non-membrane proteins interestingly showed significantly increased expression in the irradiated cells at different time points (Table 1), those were myosin, plectin, vimentin, lamin, actin cytoplasmic 2 and filamin B, which mainly connects cell membrane constituents to the cytoskeleton and maintain cell integrity [26], while histone H2A is involved in the structure of chromatin [46]. The expression patterns of these proteins are intriguing; they could potentially be targeted with the use of ligands to deliver thrombotic agents to AVM vessels. We have used Sulfo-NHS-LC-biotin in this study, which is known to inhibit cell membrane penetration [32]. Hence the presence of cytoplasmic and some nucleus proteins may be the result of radiation-stimulated surface expression. Previous in vitro studies of endothelial cells have shown cell surface translocation of intracellular proteins in response to irradiation [29, 34]. However the possibility of some biotin permeation cannot be excluded (Table 3).

**Table 3:** Membrane proteins of bEnd.3 identified in irradiated (R) and control (C) samples at 6, 24 and 48 h after irradiation, their average masses, average concentration on column (fmol) and number of replication in the MS runs; n = 6 (R); n = 6 (C)

Time point	Protein name	MW (Da)	Ave (fmol) in R	Protein replication	Ave (fmol) in C	Protein replication	Fold change (R:C)
6 h	PECAM-1	82,118.1	21.7	1	13.5	1	1.6
	Cadherin 5	88,188.2	0.01	1	6.5	1	0
	Annexin A2	38,961.4	6.4	1	6.7	1	1
	Multimerin 2	105,833.7	0.01	1	6.5	1	0
	Integrin beta 1	91,482	0.01	1	6.4	1	0
	Ras related Rab-1B	22,358.3	2.4	1	0.01	1	0
	Protein lunapark	47,785.1	13.2	1	0.01	1	0
24 h	PECAM-1	82,118.1	67.9	5	52	5	1.2
	Cadherin 5	88,188.2	49	6	16.4	6	3
	Multimerin 2	105,833.7	25.4	3	28.9	3	0.8
	Protein lunapark	47,785.1	23.2	1	0.01	1	0
	Endothelial protein C receptor	27,668.3	7.2	1	0.01	1	0
	Surface glycoprotein	72,515	0.01	1	13.4	1	0
	Annexin A2	38,961.4	9	1	9.4	1	1
	Integrin beta 1	91,482	31.7	3	0.01	1	0
	Fibroblast growth factor 8	23,891.9	19.8	1	0.01	1	0
48 h	PECAM-1	82,118.1	55.4	5	36.7	3	1.5
	Annexin A2	38,961.4	0.01	1	10.4	1	0
	Cadherin 5	88,188.2	31.1	3	18.3	1	1.7
	Multimerin 2	105,833.7	19.8	3	21.7	3	0.9
	Cell surface glycoprotein	72,515	15.8	1	0.01	1	0
	Integrin beta 1	91,482	15.1	1	0.01	1	0
	Ras related Rab 35	23,310.4	10.7	1	0.01	1	0
	Fibroblast growth factor 8	30,647.8	0.01	1	4.9	1	0

### MS^E^ data

The most extensive membrane protein up-regulation in the irradiated cells was observed at the 24 h time point. Those membrane proteins were PECAM-1, cadherin 5, integrin beta 1, endothelial protein C receptor (EPCR), and multimerin 2. At the 24 h time point, these proteins were significantly up-regulated by 3.8-, 5.7-, 14.2-, 17.7- and 3.2-fold respectively (Table 4). At the 48 h time point, PECAM-1 showed significant up-regulation in irradiated cells, by 2.3-fold, while cadherin 5 and multimerin 2 were up-regulated by 3.3- and 1.0-fold, respectively (Table 5). These results are in accordance with the iTRAQ-MS data and with the immunocytochemistry data.

**Table 4:** Membrane protein upregulation in irradiated (R) versus control (C) cells at 24 h post irradiation, their accession number, molecular weight, average concentration on column (fmol) and average concentration ratios (irradiated: control); n = 6 (R); n = 6 (C)

Protein name	Accession#	MW	Ave fmol in R	SD	Ave fmol in C	SD	Ave (R:C)	*p*
PECAM-1	Q08481	82,118.14	88.4	57	23.4	26.1	3.8	*0.019*
Cadherin 5	P55284	88,188.25	58.7	33	10.3	10.2	5.7	*0.010*
Integrin beta 1	P09055	91,481.98	26.9	16	1.9	0.2	14.2	*0.004*
EPCR	Q64695	27,668.34	35.3	18	2	0.2	17.7	*0.058*
Multimerin 2	A6H6E2	105,833.7	39.3	22	12.4	12.1	3.2	*0.033*

**Table 5:** Membrane protein upregulations in irradiated (R) versus control (C) cells at 48 h post irradiation, their accession number, molecular weight, average concentration on column (fmol) and average concentration ratios (irradiated: control); n = 6 (R); n = 6 (C)

Protein name	Accession#	MW	Ave fmol in R	SD	Ave fmol in C	SD	Ave (R:C)	*p*
PECAM-1	Q08481	82,118.14	45.0	23.0	19.2	17.9	2.3	*0.056*
Cadherin 5	P55284	88,188.25	16.8	15.7	5.1	6.5	3.3	0.136
Multimerin 2	A6H6E2	105,833.7	11.4	9.5	11.8	11.1	1.0	0.938

Since PECAM-1 was up-regulated significantly in both iTRAQ-MS and MS^E^ data at various time points in irradiated cells, future studies will investigate PECAM-1 protein as a target for AVM molecular therapy with the use of ligands that have been suggested for PECAM-1, such as α_v_β_3_ integrin, CD38, CD51 and CD61 [17, 42].

Cadherin 5 expression was also significantly up-regulated in both iTRAQ-MS and MS^E^ in irradiated cells at various time points. Therefore future work will further investigate the role of cadherin 5 in thrombosis of AVM vessels post irradiation.

Integrin beta-1 is another protein that was up-regulated significantly in irradiated cells at 24 h by 14.2-fold, post irradiation with MS^E^ analysis (Table 4). Integrin beta-1 (or CD29) is a membrane receptor belonging to the family of integrins, and is involved in cell adhesion and recognition in a variety of biological processes including embryogenesis, haemostasis, and metastatic diffusion of tumour cells [30]. The subfamily alpha 2–beta 1 (ɑ_2_β_1_) integrin serves as a receptor for collagens, laminin, and many other ligands, and it has been extensively studied as a collagen receptor on platelets [33]. Many studies suggested that ɑ_2_β_1_ is a crucial mediator of platelet adhesion to collagen in the vessel wall after vascular injury, and that this interaction is required for proper haemostasis and thrombosis [33]. In 2002 a study by Cordes et al. [4] showed that integrin beta-1 was up-regulated post irradiation in human lung tumor cells in vitro. The role of integrin beta-1 receptor in thrombosis makes it a potential protein target for investigation for the AVM molecular therapy post radiosurgery.

Endothelial protein C receptor (EPCR) or (CD201) is an additional membrane protein that was up-regulated significantly in the irradiated cell cultures at 24 time point by 17.7-fold (Table 4). This protein is a receptor for activated protein C (APC), which is a serine protease involved in the blood coagulation pathway [10]. Protein C, also known as autoprothrombin IIA and blood coagulation factor XIV, which is an inactive protein, the active form plays important role in regulating thrombosis [13]. Protein C is activated by binding to thrombin, and protein C’s activation is stimulated by the presence of thrombomodulin and EPCR, therefore activated protein C is mainly found in endothelial cells of blood vessels walls [10, 31]. Due to EPCRs role in regulating thrombosis it makes this protein a worthy candidate for investigation in AVM molecular therapy, perhaps adding APC resistance factors after radiosurgery may induce thrombosis readily in AVM vessels.

Multimerin 2 was up-regulated in irradiated cell at 24 h by 3.2-fold (Table 4). Multimerin 2 is a basement membrane glycoprotein secreted into the extracellular matrix that inhibits endothelial cells motility and negatively regulates angiogenesis [2, 20]. Some in vitro and in vivo studies suggested that multimerin 2 may reduce tumour angiogenesis and growth by interfering with the vascular endothelial growth factor (VEGF-A/VEGFR2) pathway thus making it a potential candidate in developing cancer treatment [16, 20]. While multimerin 1 in platelets plays a role in thrombosis [15], mulimerin 2 doesn’t, and there is no previous evidence of multimerin 2 response to irradiation.

In conclusion, cell surface protein biotinylation and iTAQ-MS and MS^E^ proteomics analysis successfully identified membrane proteins from murine brain endothelial cell cultures in response to irradiation. The up-regulated membrane proteins identified from this novel research are currently being investigated as potential targets for the ligand-directed molecular targeting trials in the rat model on AVM, along with the membrane proteins identified from the proteomics analysis of the rat model of AVM in response to irradiation [37], manuscript in preparation for submission).

Future study will further include irradiation-induced changes in human primary endothelial cell cultures from resected AVM tissues, using proteomic analysis. Candidate proteins then will be investigated for use in ligand-directed human vascular targeting trials to promote rapid thrombosis in AVM vessels after radiosurgery. This is especially important for patients who currently have to wait up to 3 years after undergoing radiosurgery, for their AVM to be occluded completely, while they remain at risk of haemorrhage.

## Additional files

**Additional file 1: Table S1.** Membrane proteins in all 3 iTRAQs experiments.

**Additional file 2: Table S2.** One of iTRAQ-MS raw data file.

**Additional file 3: Table S3.** All proteins identified in 2 MSEs.

**Additional file 4: Table S4.** MSE raw data for control at 6 h, one fraction.

**Additional file 5: Table S5.** Combining Tables 1, 2, 3, 4, 5 and the staining data, all in one table.
